# Alien ants spreading through Europe: *Brachyponerachinensis* and *Nylanderiavividula* in Italy

**DOI:** 10.3897/BDJ.12.e123502

**Published:** 2024-05-21

**Authors:** Enrico Schifani, David Grunicke, Andrea Montechiarini, Carlos Pradera, Roger Vila, Mattia Menchetti

**Affiliations:** 1 University of Parma, Parma, Italy University of Parma Parma Italy; 2 Institut de Biologia Evolutiva (CSIC-Univ. Pompeu Fabra), Barcelona, Spain Institut de Biologia Evolutiva (CSIC-Univ. Pompeu Fabra) Barcelona Spain; 3 University of Hohenheim, Stuttgart, Germany University of Hohenheim Stuttgart Germany; 4 Lancaster University, Lancaster, United Kingdom Lancaster University Lancaster United Kingdom; 5 Anticimex 3D Sanidad Ambiental SA, Sant Cugat del Vallès, Spain Anticimex 3D Sanidad Ambiental SA Sant Cugat del Vallès Spain

**Keywords:** invasive alien species, tramp ants biosurveillance

## Abstract

The number of known alien ant species throughout Europe has been steadily increasing during the last few decades and Italy has been no exception, with four new taxa reported in the last five years. Here, we document new data on the Asian needle ant *Brachyponerachinensis* (Emery, 1895), an invasive alien species whose first establishment in Europe was detected in the southern Italian city of Naples in 2022 and which has now been found near Lake Como in northern Italy, representing the second European record, about 730 km distant from the first. Furthermore, we report for the first time the presence of *Nylanderiavividula* (Nylander, 1846) in the country, based on specimens collected both in Rome and near Lake Como. This is at least the second *Nylanderia* species established in the country after *N.jaegerskioeldi*, first reported in 2018. Unlike *B.chinensis*, *N.vividula* is not considered an ecological and health threat in the invaded range and is already known to occur in several other European countries. While only a few introduced ants in Europe are considered serious ecological, economic or health threats, the increasing circulation of several alien species and the poor ability to swiftly track their movements and detect their establishment can render management very difficult.

## Introduction

Several species of ants have been introduced around the world, some of which are recognised as invasive species capable of generating significant harm to the environment, human activities or health (Holway et al. 2002, Wong et al. 2023). The number of alien ant species in Europe has been rising for decades (Schifani 2019) and, during the last few years, new species of particular concern have been discovered, including the Asian needle ant *Brachyponerachinensis* (Emery, 1895) (Menchetti et al. 2022) and the red imported fire ant *Solenopsisinvicta* Buren, 1972 in Italy (Menchetti et al. 2023, Menchetti et al. 2024) and the little fire ant *Wasmanniaauropunctata* (Roger, 1863) in Cyprus, France and Spain (Espadaler et al. 2018, Espadaler et al. 2020, Demetriou et al. 2022, Blight et al. 2023, Pradera and Espadaler 2024).

Italy hosts over 20 alien ant species (Jucker et al. 2008, Schifani 2019, Schifani 2022, Menchetti et al. 2022, Menchetti et al. 2023, Schifani et al. 2024). The establishment of some of these is to be considered doubtful given the absence of further records, while no monitoring programmes for these processes exist (Jucker et al. 2008). While most of them likely fail to establish viable populations, the number of species arriving through international trade is substantial according to the few available data (Jucker et al. 2008, Wong et al. 2023). Biosurveillance capabilities are still unable to cope with this phenomenon, because detections are typically late as compared to their establishment and are not the result of targeted efforts (Blight et al. 2023, Menchetti et al. 2024).

Here, we report on the presence of a new alien ant species in Italy, the crazy ant *Nylanderiavividula* (Nylander, 1846), already widespread in Europe (Guénard et al. 2017). The genus *Nylanderia* counts several successful alien species introduced around the world, including many taxonomically difficult groups and some supercolonial species (Williams et al. 2020, Williams and Lucky 2020). Amongst these, so far only *Nylanderiajaegerskioeldi* was known to have been established in Italy, becoming widespread in Sicily and its neighbouring islands (Schifani and Alicata 2018, Schär et al. 2020 - records of unidentified *Nylanderia* morphospecies in these papers should also be attributed to *N.jaegerskioeldi* (Mayr, 1904) according to a more recent investigation of the voucher specimens). A second species, *N.bourbonica* (Forel, 1886) was at least once intercepted at a cargo harbour (Jucker et al. 2008).

Furthermore, we report on a population of the Asian needle ant *Brachyponerachinensis* in northern Italy, at a site about 730 km north-west of Torre Annunziata, near the city of Naples, the only locality where the species was known in Europe (Menchetti et al. 2022). The new site of *Brachyponera* in Italy was first reported on the platform iNaturalist.org, after which specimens were collected to identify the ants at species level. While currently not listed amongst the species of concern by the European Union, *B.chinensis* is certainly amongst the few alien species which may be considered as an ecologically threatening invader, as well as a public health threat due to its stinging abilities (Nelder et al. 2006, Guénard and Dunn 2010, Suehiro et al. 2017, Guénard et al. 2018).

## Materials and methods

Worker specimens were collected in ethanol and stored in the authors' personal collections and voucher specimens were deposited at the Institut de Biologia Evolutiva (CSIC-Univ. Pompeu Fabra), Barcelona, Spain. They were examined under a stereoscopic microscope with up to 180x magnification, while photos were taken using Canon MP-E 65mm f/2.8 1–5x macro lens and measurements were taken using the software ImageJ (Schneider et al. 2012). Identification was performed using the taxonomic information provided by Yashiro et al. (2010), LaPolla et al. (2011), Kallal and LaPolla (2012), and Salata et al. (2018). Maps were plotted using the R package "ggmap" (Kahle and Wickham 2013) using Stadia Map and Stamen Design.

## Results

**Figure 1.**
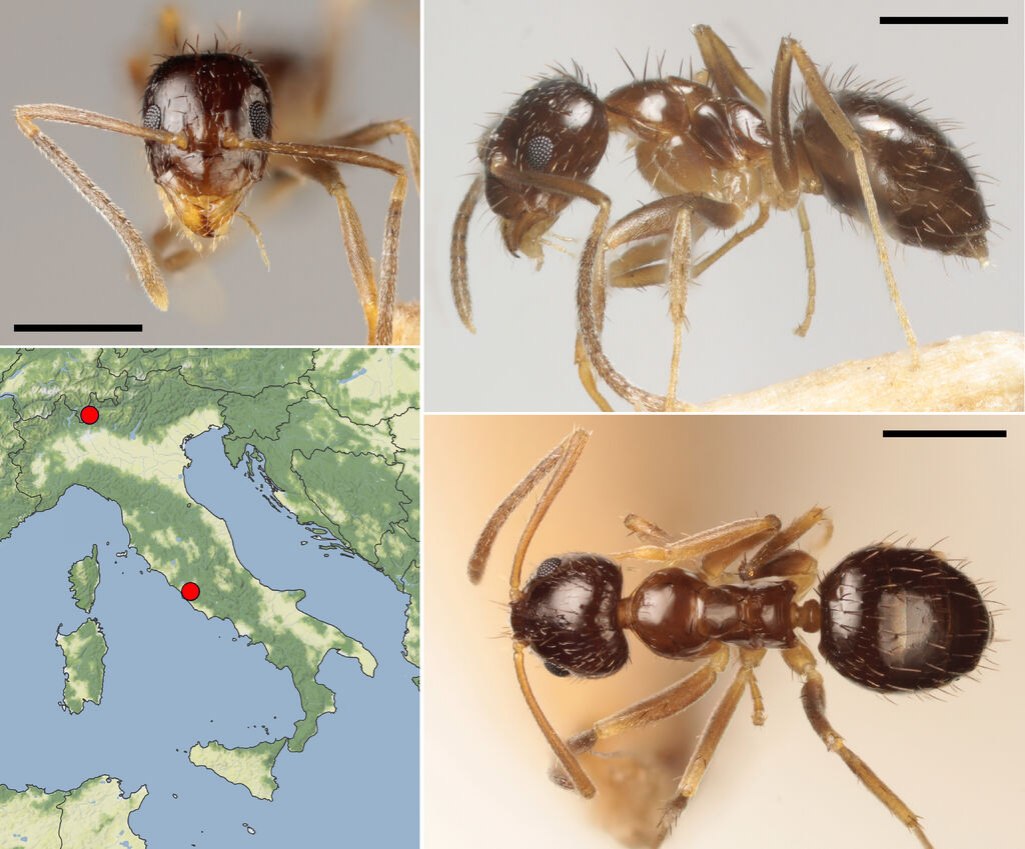
Head, lateral and dorsal view of a *Nylanderiavividula* worker collected in Rome by Carlos Pradera (scale bars: 0.5 mm) and distribution map of the first Italian records of this species (map from Stadia Maps - stadiamaps.com and Stamen Design - stamen.com).

**Figure 2.**
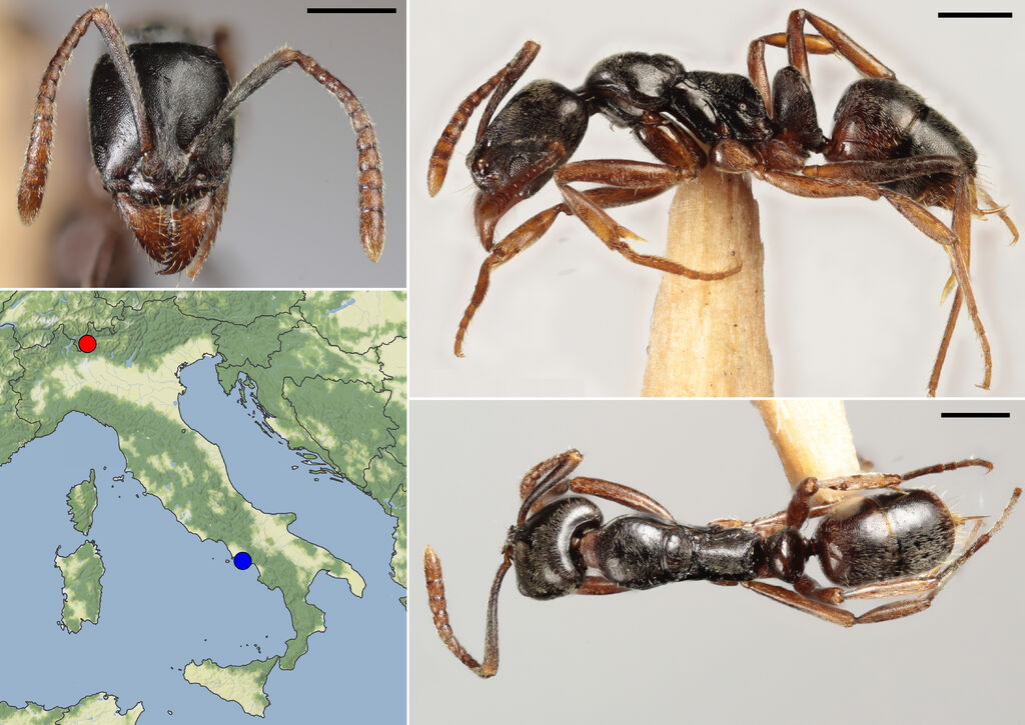
Head, lateral and dorsal view of a *Brachyponerachinensis* worker collected near Lake Como by Andrea Montechiarini (scale bars: 0.5 mm) and distribution map of the Italian records of this species: in blue, the site near Naples reported by Menchetti et al. (2022), in red the new Lake Como site. Map from Stadia Maps (stadiamaps.com) and Stamen Design (stamen.com).

**Figure 3.**
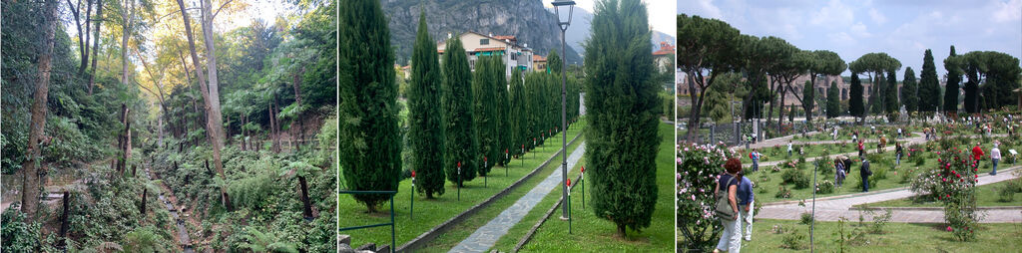
From left to right, the collecting sites of Villa Carlotta (where both *B.chinensis* and *N.vividula* were collected), Griante and the Roseto Comunale in Rome (in both of which, only *N.vividula* was found).

Specimens of *N.vividula* were identified from a site in Rome (Latium) and two sites near Lake Como (Lombardy) (Fig. 1), while *B.chinensis* was collected in one of the same sites near Lake Como (Fig. 2). In all three sites, foraging workers were abundant in garden areas with exotic plants (Fig. 3). Data of the new records are listed in Table 1.

## Discussion

International trade, especially of plants, is considered the leading cause for the high frequency of ant introduction across the world (Bonnamour et al. 2023, Mwebaze et al. 2023). Furthermore, bridgehead effects, in which an invasive population becomes the source of additional invasions through secondary introductions, appear to have a key role in the spread of invasive ants (Bertelsmeier et al. 2018). Lastly, climate change may significantly alter the potential distribution of many invasive ant species (Bertelsmeier et al. 2014).

In just the last five years, six new alien species have been discovered in Italy: *B.chinensis*, *Hypoponeraergatandria* (Forel, 1893), *N.jaegerskioeldi*, *S.invicta*, *Tetramoriumlanuginosum* (Mayr, 1870) and now *N.vividula* (Schifani and Alicata 2018, Menchetti et al. 2022, Menchetti et al. 2023, Schifani et al. 2024). While *H.ergatandria* is a cryptic species of recent recognition and may potentially have been in Italy since at least several decades (Schifani et al. 2024), the remaining species could be introductions of the last 20-30 years, even considering the chance of a long lag time before detection (Menchetti et al. 2024).

The presence of *Nylanderiavividula* in the Mediterranean and Europe has long been documented and seems restricted to human settlements. It is likely not a particularly threatening species as no ecological damage or serious pest status is reported from any country - although it can locally become numerically prevalent over other ants as observed in Rome. It is native of the New World, but has been introduced as far as Eastern Asia, Papua and the Afrotropics in addition to the Western Palearctic (Kallal and LaPolla 2012, Guénard et al. 2017). In Europe, Spain (mainland and Balearic Islands) was so far the only country where it had been reported as established outdoors (Gómez and Espadaler 2006, Martinez Ibanez et al. 2007), while it has been reported indoors from a large number of others: Croatia, Denmark, Finland, France, Germany, Ireland, Netherlands, Norway, Poland, western Russia, Serbia, Sweden, Ukraine and the United Kingdom (Guénard et al. 2017). However, it is also worth noting that, despite containing several invasive species, the genus *Nylanderia* only recently drew significant taxonomic attention and misidentifications may have been common in the past (Blatrix et al. 2018).

On the other hand, *B.chinensis*, which is a strictly predatory species unlike most other alien ants in Europe, can be ecologically damaging at least in temperate forest ecosystems and cause ecological cascade effects by displacing native ants and disrupting ant-plant mutualisms (Bednar and Silverman 2011, Rodriguez-Cabal et al. 2011, Suehiro et al. 2017). Together with the recently discovered *S.invicta*, it is also capable of delivering painful stings to humans (Nelder et al. 2006). This species is native to parts of Eastern Asia, where it is now present in mainland China, Taiwan, the Korean Peninsula and Japan and was notably able to spread over 17 USA States since its first introduction in the 1930s (Guénard et al. 2018). More recently, it was introduced along the eastern coast of the Black Sea in Georgia and Russia and the only presence of *B.chinensis* in Europe and the Mediterranean was so far represented by the Italian record near Naples by Menchetti et al. (2022). A reported interception in the German city of Hamburg under the name "*Ponerasolitaria*" and dating back to 1900, is considered dubious (Menchetti et al. 2022). While only a flying male was initially collected in Torre Annunziata (Naples) in July 2020, additional males were found under the same conditions in June 2022-2023 (Vincenzo Gentile, pers. comm.), further confirming the establishment of the species in the area - while it remains unclear where the colonies are located as no targeted monitoring efforts were conducted to the best of our knowledge.

At the moment, both species are exclusively known from urban areas and gardens in Italy, which is where most alien ants in southern Europe are confined (Schifani 2019, Demetriou et al. 2023, Demetriou et al. 2023b). Botanical gardens, hosting exotic plants, are often hotspots of alien soil invertebrates (Schifani and Alicata 2018, Mori et al. 2021). It is notable that Como Lake is characterised by a significantly warmer microclimate than most of northern Italy, often favouring biological invasions (Pautasso 2013). However, both the first discovery of *N.vividula* in the country and the appearence of *B.chinensis* at a very distant site from its previously known distribution highlights how alien ants are often able to spread undetected and how opportunistic sampling remains the prevailing mechanism through which they are eventually discovered (Blight et al. 2023, Menchetti et al. 2024).

## Figures and Tables

**Table 1.** List of the new alien ant records presented in this study.

**Species**	**Region**	**Site**	**Lat., Long.**	**Collecting date**	**Collector**
*B.chinensis*	Lombardy	Villa Carlotta, Como Lake	45.98763, 9.23261	9 Sept 2023	D. Grunicke
*B.chinensis*	Lombardy	Villa Carlotta, Como Lake	45.98763, 9.23261	28 Sept 2023	A. Montechiarini
*N.vividula*	Latium	Roseto Comunale, Rome	41.88554, 12.48344	7 Jun 2023	C. Pradera
*N.vividula*	Lombardy	Griante, Como Lake	45.99358, 9.23433	28 Sept 2023	A. Montechiarini
*N.vividula*	Lombardy	Villa Carlotta, Como Lake	45.98763, 9.23261	28 Sept 2023	A. Montechiarini
